# New York Odontological Society

**Published:** 1892-02

**Authors:** 

**Affiliations:** New York Odontological Society


### Reports of Society Meetings.

---

### NEW YORK ODONTOLOGICAL SOCIETY.

A REGULAR meeting of the New York Odontological Society was held Tuesday evening, November 17, 1891, at the New York Academy of Medicine, No. 17 West Forty-third Street.

The President, Dr. William H. Dwinelle, in the chair.

*The President.*—Gentlemen, we are fortunate in having for our essayist this evening one of Philadelphia's most prominent practitioners, who will take us into his confidence concerning the rules and regulations he has formulated for the government of his practice.

I take pleasure in introducing Dr. Louis Jack.

(For Dr. Jack's paper, see page 81.)

### DISCUSSION.

*The President.*—Gentlemen, Dr. Jack's address is before you for consideration. We would like to hear from Dr. Howe.

*Dr. Howe.*—The presentation of the subject has been such that I have no doubt, as the discussion proceeds, it will be more efficiently dealt with than I feel adequate for this evening. In one sense there is nothing of more importance than that we should learn order; to husband our physical resources, make the most of ourselves in our practice, and make the most we can of our practice. I think Dr. Jack's presentation of the subject has been so forcible that it must have impressed itself upon the minds of all present. I feel that some of the methods presented will be of great service to me. I would like to ask Dr. Jack to explain a little more fully the use of what he calls his monthly index. If he can add a word or two on that subject, and allow the book to be passed around, I think we would all learn something from that. It was not quite clear to me how it was to be used.

*Dr. Jack.*—To make this subject clear I will state the routine of the moment when a series of cases are completed. The statement is made to the patient that an appointment for examination should be renewed at a proper period, and the question is distinctly asked,

Whether this shall be done? This is because at one time when such an appointment was sent, the patient replied by returning the card accompanied by his personal card, on which was written, "Mr. —— has no appointment with Dr. Jack." The cut was so keen that the language of the examination-card was changed to read, "Mr. or Mrs. —— has, in agreement with previous arrangement, an examination appointment at ——." It is seen the card carries the agreement on its face. The above arrangement is carried out by the secretary entering the name, address, and kind of appointment required on the monthly index at the proper place. In this manner, as the time passes, the names of nearly all the regular patients find their places on the monthly index, and without much effort the preliminary engagements become evenly distributed.

As the time approaches, the appointments are carried forward into the engagement-book, from which the notifications are made a week in advance of the date given.

As there are six hours of each day allotted for operative service, it will be observed that three appointments for examination will be sufficient to fill completely the time, if to each patient is given two single-hour or one two-hour engagement, therefore about eighty persons arranged for any given month occupy the whole of the time. When patients are periodically examined and the cases carefully attended to, the average service required by each one becomes less than two hours twice per year. Some may require nothing done; some one hour; others several hours. Upon this average the whole arrangement and allotment of time is based.

*Dr. Northrop.*—In making those appointments, you say, "Within three or six months it will be necessary to make an examination of your mouth," and you enter it on your book. Is it simply for an examination, or do you make provision for further time?

*Dr. Jack.*—As I have stated, we make other appointments on the book. There are, however, some persons who rarely require operations, and the memorandum then will call only for an examination.

*Dr. Northrop.*—At the time you finish, you consider their teeth in perfect order, and yet you say they should have their mouths examined. Do you anticipate further trouble, or is it done merely as a precautionary measure?

*Dr. Jack.*—It is assumed that the large majority of people require some service each six months and some persons even more frequently.

*Dr. Northrop.*—You anticipate that two hours is sufficient?

*Dr. Jack.*—That appears to be an approximate average, and is in agreement with the statement I made this evening, that three examinations each day, and six one-hour, or three two-hour appointments fill out the day. No patient is allowed at the same time more than two one-hour appointments, or one two-hour appointment; but if they have much service to be performed, they are placed upon an emergency list, which the secretary keeps, of people who need much service. Such persons, also, have the advantage of receiving the hours which may be opened.

*Dr. Kingsley.*—I should like to ask Dr. Jack in reference to charging for the time of examination?

*Dr. Jack.*—I charge for the examination according to the time occupied.

*Dr. Kingsley.*—Suppose it be fifteen minutes?

*Dr. Jack.*—My usual time is ten minutes. Sometimes it requires twenty. If I cannot thoroughly examine a mouth in ten minutes, the remaining portions are noted, and the examination is completed at another time.

*Dr. Kingsley.*—And that charge is one-sixth of an hour?

*Dr. Jack.*—It is; I invariably charge for the examination appointment. In the front of the book will be found the different forms of stationery that are used. A page of the chart-book of examinations, which is kept before me each day, and the day slip, which my secretary makes out in the morning, with the names of the patients upon it, opposite the given hour. This slip is kept behind me on a lectern, and, as I get through with the case, I mark what I have done, and the symbol of the charge. These symbols are carried forward by the secretary into the appointment-book, and become the first entry. Another form will also be found which is for the use of any one who may happen to call and not be able to get my attention, or who may come at a time when I am absent. The person fills out this slip with an expression of his wants.

PLEASE WRITE BELOW—NAME, ADDRESS, AND PURPOSE OF CALL. THE MESSAGE WILL HAVE EARLY ATTENTION.

*Name,*..........

*Address,*..........

*(Purpose)*

*Dr. Jarvie.*—I would like to ask Dr. Jack, as he reserves only the hour between twelve and one for examination and short treatments, how he attends to children who are at school during the day, and men who are at business, as it must be inconvenient for them to call at that time?

*Dr. Jack.*—I have carried on this system so resolutely and for so many years that my people have all fallen into the habit of it. They know they cannot see me except at a certain time, and the overpressure has been so great that I do not care to be troubled by people who will not conform to the regulation, which is in the interest of all concerned. I would say, however, that if any one should come to my office in pain, I must find relief for them, as this is a necessity which knows no law. I then ask to be excused from my patient to give the necessary relief; but when a practice is conducted in the manner presented, it is of comparatively rare occurrence that any one will appear in great suffering. The ordinary inconveniences can be deferred by any one until the hour of twelve-thirty. The hour from twelve to one is divided, the first half of it into appointments for examination; in the other half of it small appointments are made, and, as I have stated, unavoidable calls are received, which leaves half an hour, from one to one-thirty, for rest and lunch. Occasionally the time for lunch is taken away by the necessity of attending to some patient who may call at twelve-thirty in a condition requiring immediate treatment. This, however, is one of the incidents of the life of every physician.

*The President.*—I feel as though I could hardly forgive Dr. Jack for not reading me his lecture fifty years ago. Dr. Jack's address is a very fine exemplification of Milton's saying that order was heaven's first law. Are there any further remarks? Dr. Jack is very kind and communicative.

*Dr. Jarvie.*—In my own practice cases occur which I term "emergency cases." For instance, a tooth is broken, or some accident has occurred that demands immediate or almost immediate attention, and perhaps requires considerable time. I do not quite understand whether Dr. Jack fills his book so closely as to leave no room for this class of cases.

*Dr. Jack.*—My book is generally filled very far ahead. Those people you speak of generally come at twelve-thirty. They are unavoidable calls. If a person comes suffering, I always attend to him.

*Dr. Jarvie.*—I mean such cases as this: A person, in biting a chocolate caramel, the other day, broke off a lateral incisor. A few minutes would not be sufficient to attend to that, and yet it must be attended to immediately. The patient must be relieved of the deformity within twenty-four or forty-eight hours. Do you put one of your other patients off?

*Dr. Jack.*—I will answer that. I might state that even when one endeavors to keep all the time actually filled, the appointment-book is constantly changing. The secretary watches for those changes, and she keeps a list of people with whom to fill opened hours, so that the book, while it may be filled far ahead, is not so inflexible as may appear, because there are circumstances which compel people to consider our appointments of less importance than some other things to which they have to attend; but the immediate want, if it is a case of suffering, is attended to then and there. If the tongue is being scratched, the chisel is used, even if it delays an appointment or takes from me my rest and lunch.

*Dr. Jarvie.*—The question in my own case is how to relieve myself of the great pressure from a superabundance of patients and at the same time to do justice to those for whom I work, by not being called away from operations any more than is absolutely necessary. I have not, however, adopted such a rule as Dr. Jack has.

It may be owing to the situation of the large village of Brooklyn, but I find that I have the most calls when gentlemen are on their way to business in the morning, and directly after the schools are out, and I do not know what I would do with my school children, if I did not see them at that time. Such an arrangement as Dr. Jack describes, with the aid of a secretary, relieves one of a great amount of labor, and as it is a service that can be done just as well by some one else as by the dentist, that time is saved for him to make operations in the mouth.

I do not think Dr. Jack is wise in one particular; he sometimes goes without his lunch. I never do that, and it only goes to prove that one can arrange his time if he so wishes. I think sometimes my time is about as much occupied as any one's can be, and yet I do not vary more than five minutes in taking my lunch at one o'clock, and from one to one-thirty I am not to be seen by any one. I am away from my office to all intents and purposes, as far as my patients are concerned. That half hour is mine, and I find that by doing so I keep myself in much better condition than I otherwise could, and it is necessary for a dentist to be in good condition all the time to do justice to his patients. It is a very important question to a dentist how he may economize his time so that he may earn the more in the seven or eight hours that he has to work, and that his patients may be subjected to the necessary discomfort or pain as short a time as possible, and it would seem, from what we have heard to-night, that Dr. Jack has solved that problem in the best interests of both patient and dentist.

*Dr. Hodson.*—I would like to ask Dr. Jack what course he pursues with patients who are late in keeping and forgetting appointments, or not keeping them through inability,—whether he charges his full fee, and in case of any change of appointment, how much time he requires?

*Dr. Jack.*—I would state to the gentlemen present that I almost invariably charge for losses of time. I do not charge for the loss of time by the breach of an examination appointment, because there have been instances in which patients have taken that roundabout way of ceasing to continue my services, and for that reason I have not been in the habit of charging for the loss of that time, and also because this hour is filled by the various calls, and therefore there is no actual loss. For regular appointments for service which are broken a charge is usually made, unless notice is given, and that charge I might state is not always for the full value of the time, though in the large majority of instances I charge what may be called the *cost* of the time. The cost of the time is one's annual expenditures, divided by the number of working hours in the year, and if this be charged, the loss of that hour is not a deduction from the other hours of the year. Sometimes, however, the failure to keep an appointment is of real benefit, as the time may be used for needed rest; but in the large majority of instances, the loss of an hour is more than the loss of the whole value of the time, because it impairs one's morale by breaking up what may be called the "working spirit" of the day, and the whole day may be impaired by the loss of an hour in the morning. One can hardly define what that influence is, but it is a potent one, and most operators must have frequently experienced it.

*Dr. Hodson.*—Dr. Jack forgot to answer how long previous to the appointed time he requires notice of change, and I would be pleased to learn whether he charges for appointments broken unavoidably.

*Dr. Jack.*—On my cards I state "reasonable" notice. Every properly-constituted person will give such notice as would enable one to fill the hour. If a man should send me five minutes notice, I would charge him as much as my conscience would allow; but if I am given one hour's notice, I try to fill the time. The usher is sent out to people whom we have near us, who would be willing to come at any time. If there be two hours' notice, then it is not difficult to fill the opened time.

*The President.*—We would all like to hear from Dr. Perry.

*Dr. S. G. Perry.*—I think it is evident that I was justified in urging Dr. Jack to write on this subject. Perhaps in no way can I discuss his paper so well as by relating my own experience and describing my method of conducting my own practice. If there has been any order or system in my office, for many years past, I think I may say I am indebted mainly to Dr. Jack for it. I have been fortunate enough to know him for many years, and I have adopted many ideas which he has explained here to-night, and have found them of great benefit to me. In some respects I have carried them as far as he has; in others, not. Many years ago he kindly sent me a book for recording the names of patients to be sent for, such as he has shown here this evening; but I confess I did not have the courage to fully carry out the plan. I found it to be more burdensome than I expected it would be, and finally abandoned it almost altogether, feeling that I must, after all, put the responsibility of returning for examinations upon the patients themselves. Yet I now have, and have had for years, quite a large number of patients whom I send for regularly for examinations. They are known to my secretary, and are sent for without any effort on my part; but when it comes to sending after all my patients, as I presume Dr. Jack does, I have receded from that. The matter of keeping an exact chart is one which interested me greatly when I began practice, and for many years I made an exact record of every operation I performed, including temporary ones, but within the last few years I have reduced the number of charts kept to those of more important operations, and operations on the roots of teeth, as well as the conditions of teeth where the pulps are nearly exposed.

In the matter of organizing my practice so as to make the most of every moment, I adopted years ago Dr. Jack's system of asking persons to come at certain times for examinations, consultations, etc. My hours for that purpose are from twelve to one and from four to four-thirty. The last-named hour is for children who come from school, and for business-men. But the hour from twelve to one is given over more generally to the public. It has come to be known as the hour when I can be seen, and it is not uncommon for patients to come long before that hour in order to be first. There is more nervous wear and tear on the system in these consultation hours than in any other. I aim to make these consultations prove profitable, for while I do not make a charge for a patient who comes in for a moment or two, if they sit in the chair for several minutes, I feel justified in charging them for it. I try to get my luncheon at one, but it is oftener a quarter past one. The first afternoon patient comes at one-thirty, and I try to be on time to the minute. We live in a hurried age, and more and more does the question of economizing time become of importance; no people think their time is of greater importance than those of New York City, and there is probably no place where the practice of dentistry is as hard. I can only say in the most emphatic terms that I have been under great obligations to Dr. Jack for encouraging me to adopt his plan of asking patients to come for consultations only at a fixed hour, because it has given me for the working hours between nine and twelve and half-past one and four that peace of mind which can never be known in any other way. I think I should have broken down before this, but for having the courage to say that I cannot be interrupted between nine and twelve or from one-thirty to four. I adhere to that almost invariably.

In a case of pain, however, I see a patient promptly, as that is a necessity that knows no law. My secretary and a maid are always on my office floor. The maid attends the door and ushers patients in and out of the operating-room, the secretary assists me about the chair, takes charge of my instruments, and writes my letters from dictation. I try to be very strict, however, about dictating while at work. For many years I have realized that there are few patients who will tolerate the distraction of attention that will come from dictating letters while operating. There are cases of children and others where one's own tact will tell him that he is justified in carrying on a double action of the mind; but there is great danger in it, and I try to avoid the danger. Without the aid of my secretary and occasional dictation, I should be almost stranded. I seldom step out of my office from the time I go there until I get my lunch. It is the maid's duty to ask the patients in and usher them out. It is the secretary's duty to make the appointments. I say to her, "Give this patient half an hour," or an hour, as the case may be. This is done in another room, leaving the office free for the next patient. The length of time given is invariably written out on the appointment-card and in the appointment-book. I never make an appointment, rarely see my appointment-book, and almost never know who comes next until the door is opened and the patient is ushered in. There is always a list of waiting patients to be sent for, and if a letter or telegram comes stating that a patient cannot come for a certain hour, it is easy to send out for one to fill the place. It is not often, therefore, that I have to charge for lost time, but sometimes I do.

It seems to me that this question of systematizing and economizing one's time is of supreme importance; there is no question that should be studied and kept before the mind as this should be. It is due to the dentist, to his family, and to the patient. One cannot perform good service if he is often disturbed. I know of nothing that will injure a dentist so much as to leave a patient in the chair, and go out to attend to other business; and I know of nothing more destructive to the temper of patients than to have the rubber dam nicely adjusted, and then have the operator called away to discuss the question of the origin of the universe and the natural tendency of things! I notice patients are more and more jealous of time. Even little children have their engagements,—their dancing classes, their French, their German, and classes of all kinds. Ladies have their engagements at lectures, in classes, for music or for various other things. It is a telegraphic, electric age, and one must be most prompt and exact in all they do. Above all, one must be faithful in keeping appointments with their patients. I have my instruments in such a condition that they are always ready for use; many are numbered and placed in numerical order. After being used for one patient, they are cleansed and sterilized, so that there is always a bright and new case of instruments for the new patient. My secretary is so efficient and orderly that I can find without an instant's loss of time just what I want. Every instrument is polished and in perfect order and in its proper place. That gives me more mental comfort and more courage than anything else about my office. I think it has a good effect on the minds of the patients also. If a dentist's shoes are not well blackened, and he be not neatly dressed, patients will give him the credit of being untidy; they will say if he does not do things neatly generally, he will not be likely to do nice dental work. I believe that applies to all one's surroundings. Ladies who know nothing about dental operations can instantly tell if the rooms are well kept, and if the operator works with system and order.

*Dr. Howe.*—What did Dr. Perry mean by saying it was dangerous to dictate while one is working?

*Dr. Perry.*—The danger is that of making a bad impression on the patient, who will mentally say, "I would rather have him thinking about my work. If he would not worry about his letters he would be more careful. He is getting near the nerve; he will be on it in a moment!" There is nothing so effective in the management of a dental practice as to feel for the patient; to put yourself in his or her place. And particularly is this true of little children,—bless their dear little hearts! Self-abnegation at the operating-chair will attract and hold patients to that extent that life will in time become a burden. If real sympathy lies deep and warm in the heart of the operator, it will invariably be felt by the patient, and without the use or waste of idle words.

*Dr. Kingsley.*—I was unfortunate in not hearing the beginning of Dr. Jack's paper, but what I did hear was certainly filled with many admirable things. There is one thing particularly that I should say amen to, and that is where he pays tribute to a competent secretary. He has only changed the language of my own views as I expressed myself on that very subject about ten years ago. I think I was the first one who had ever paid the tribute that is justly due to a lady secretary. I have never changed my mind. I would emphasize it more strongly if I could, and I was much gratified at hearing Dr. Jack use exactly the same ideas.

There was a matter with reference to which I would like to talk. I do not know whether I misunderstood Dr. Jack or not, but I believe he said he bases his charge for operations upon the time employed. I once thought the same way, and I thought it was not only a good thing but the best thing; that if I gave my time for one hour I must be paid for one hour. And so for years; but by and by I began to recognize that I was doing a great many more important things for some patients, which caused more strain upon my brain than in another hour, and that the value of the service was not to be measured by the length of time expended upon it. I gradually began to think that I was degrading my profession to the level of a day laborer, charging for so many hours a day, or for so many parts of a day, per hour. To-day I read of a strike among the scene-shifters of the theatres, which had been compromised upon a basis of fifty cents an hour. I thought to myself, Why, this is exactly what I used to do, only I did not charge as low as fifty cents an hour. I thought, when Dr. Jack was reading his paper, Is all of dentistry filling teeth by the hour? Because, then, it comes to the mechanical operation of stuffing metal into a hole in the tooth per hour. Is that all there is of dentistry? We know it is not, and if it is not, the system is a bad one. Again, I think Dr. Jack conveyed the idea that it is hardly fair to charge one person one fee and another person another for what appeared to be the same work. That is the true commercial idea, of course. If I go to one store and buy a yard of Merrimac print for nine and one-half cents a yard, and another person buys the same print at my elbow for nine cents a yard, I complain. It is not fair. That is the commercial idea. But does it apply with us? I do not think so. Some time ago I had to treat one of the family of a lawyer who, to my personal knowledge, had received, for what seemed to me a limited service in his profession, fifty thousand dollars for his fee. In another case he had received a great deal more, and in many other instances probably a great deal less. The case required regulation of the teeth, and it was carried on—no matter how long. When the work was completed it was important in that the child had received a great deal of benefit, having been actually deformed before; now she was re-formed,—that is to say, the deformity was entirely removed, and the whole expression of the face was changed. The value to the child and to the parents was undoubtedly very great. The work performed had been one of only such strain upon me, so far as my inventive faculties were concerned, as a great many other things for which I have received only a very small fee. When the work was completed I sent in a bill. I had never seen the father. I sent in a bill which was more than twice as large as any fee I had ever charged. My secretary said to me that I was making a very grand mistake. I said, "How so?" She answered, "Because you never have charged before any such fee for the length of time devoted to a similar performance." I said, "No, I have not; but I know the father of that child is a professional man; he looks at the service I have performed from a totally different stand-point from a man who is a laborer, or who is selling calico at a profit of ten per cent. upon the cost. He looks upon the service as a professional service which involves brain work, and looks upon it as a valuable work, too. When he makes a charge he makes it from that stand-point. My brains are worth as much to me as his are to him, and I intend to view this thing from his stand-point,—from the stand-point of a professional man." A few days afterwards the gentleman's card came in to me. He greeted me most effusively, and said he wanted to come in a long time ago to thank me for the great service which I had rendered his child. That no amount of money could compensate him for the pleasure I had given him. There was a check, and also an appointment from him which brought me a large amount of money. Gentlemen, you say it is all right to take all the operations in dentistry on the same plan? Dentistry is not simply plugging teeth with metal fillings and polishing them off, and charging for a day's labor.

It is something more than that, and, when it is, that should be taken into consideration.

*Dr. S. C. G. Watkins.*—This is certainly a very enjoyable meeting. It seems a most important factor in the life of a successful dentist that he should be able carefully to economize his time, and handle easily patients of different temperaments. This is a life-long study for any dentist.

Much has come home forcibly to me this evening, and I feel that if we were able to carry out the principles presented in this paper we would all live longer. The doctor has worked his practice up to so extreme a point, as regards the economy of time, that few of us can expect to attain to it. In my practice, and perhaps in that of the majority, I presume it would be altogether impracticable to follow Dr. Jack's methods in every particular, but he has given us many valuable ideas, and for every one we are able to adopt benefit will come, I am sure.

*Dr. Northrop.*—I do not know that I have anything to add to what has already been said. I think the saving of time to a dentist is a great consideration, and the person who can save time saves his strength. I was very much pleased with Dr. Jack's paper. I was in Dr. Jack's office a number of years ago, and appropriated some of his ideas. As the result of that visit I have taken the hour from twelve to one o'clock for examinations and consultations, and from one to one-thirty for my lunch, and since this innovation I have had my regular operations broken into probably as little as any other man, and I think I have been able to do more work than I did before. I recollect very well, before I went to see Dr. Jack and learned how he divided his time, I had, on one occasion, a patient who had an appointment for an hour and a half, and because of the frequent calls and interruptions, I was able to give only about twenty minutes labor to that patient. The rest of the time was lost. I did not complete the operation I intended, and I was disappointed. Since I have made the above change it has been greatly to my satisfaction, and I think to my patients also.

*Dr. Brockway.*—There is one aspect of this question concerning the conduct of practice, of which I will speak, as it has been alluded to several times,—that is, the control of patients and getting them to do as we wish. I am satisfied that such men as Dr. Perry or Dr. Jarvie, or others who have spoken here, can do just what they please with their patients. The world is full of people who want the services of these men, and they are willing to make almost any sacrifice to secure such service. They will come if required to on certain days of the year, or on any hour of the day, or of the night even. These patients cannot be driven away by any rigidity of rules regarding the conduct of practice. I have listened to Dr. Jack's paper with a great deal of interest, and I only regret that he had not written it years ago. The matter of economizing time is of the first importance. That is where so many dentists fall short. They have enough to do, but their time is so broken into and divided and frittered away by lack of system and order that at the end of the day they find they have accomplished very little.

*Dr. Perry.*—Here is a little device which Mr. Hodge brought to me from San Francisco,—a device invented by Dr. Hatch, and which I have used for some time,—for holding the rubber dam on the labial and buccal surfaces of the teeth. It opens and closes by turning a screw, and it will stay firmly where it is placed. The two points of bearing are opposite each other. My associate, Dr. Woodward, with his usual ingenuity made some changes in this device, by which it is possible to easily change the points of bearing fitting it for use on teeth from which the gum has receded on the buccal and lingual sides. There are three of his devices here; two are quite alike, and the third one, which has the same idea in the main, has some slight modifications. It has three bearings, like a three-legged stool which will stand evenly on an uneven floor. The adjustment can be made so that the device can be held perfectly steady. The points of bearing can be made directly opposite each other, or it can be opened so as to have the points of bearing very far from being opposite. This little device I consider now to be almost invaluable. I should hardly know how to get on without it.

A unanimous vote of thanks was tendered Dr. Jack by the Society.

Adjourned.

S. E. Davenport, D.D.S., M.D.S.,
*Editor New York Odontological Society.*

